# How a thrombectomy service can reduce hospital deficit: a cost-effectiveness study

**DOI:** 10.1186/s12962-022-00395-8

**Published:** 2022-11-04

**Authors:** Iris Q. Grunwald, Viola Wagner, Anna Podlasek, Gouri Koduri, Paul Guyler, Stephen Gerry, Sweni Shah, Horst Sievert, Aarti Sharma, Shrey Mathur, Klaus Fassbender, Kaveh Shariat, Graeme Houston, Avinash Kanodia, Silke Walter

**Affiliations:** 1 TIME, Imaging Science and Technology, University of Dundee, Dundee, DD1 4HN UK; 2 Cardiovascular Centre, 60389 Frankfurt, Germany; 3 Department of Neurology, Saarland University Clinic, Homburg/Saar, Germany; 4 Rheumatology, Mid and South Essex NHS Foundation Trust, Southend-on-sea, Essex, SS0 0RY UK; 5 Stroke Medicine, Mid and South Essex NHS Foundation Trust, Southend-on-sea, Essex, SS0 0RY UK; 6 Centre for Statistics in Medicine, Botnar Research Centre, University of Oxford, Oxford, OX3 7LD UK; 7 Physiotherapy Department, Mid and South Essex NHS Foundation Trust, Basildon, Essex, SS0 0RY UK; 8 Clinic for Neurosurgery, Kantonsspital Winterthur, 8400 Winterthur, Switzerland

**Keywords:** Acute stroke, Thrombectomy, Cost-effectiveness, Health economics, Matched-pair analysis, Length of stay, Patient-level costing

## Abstract

**Background:**

There is level 1 evidence for cerebral thrombectomy with thrombolysis in acute large vessel occlusion. Many hospitals are now contemplating setting up this life-saving service. For the hospital, however, the first treatment is associated with an initial high cost to cover the procedure. Whilst the health economic benefit of treating stroke is documented, this is the only study to date performing matched-pair, patient-level costing to determine treatment cost within the first hospital episode and up to 90 days post-event.

**Methods:**

We conducted a retrospective coarsened exact matched-pair analysis of 50 acute stroke patients eligible for thrombectomy.

**Results:**

Thrombectomy resulted in significantly more good outcomes (mRS 0–2) compared to matched controls (56% vs 8%, p = 0.001). More patients in the thrombectomy group could be discharged home (60% vs 28%), fewer were discharged to nursing homes (4% vs 16%), residential homes (0% vs 12%) or rehabilitation centres (8% vs 20%). Thrombectomy patients had fewer serious adverse events (n = 30 vs 86) and were, on average, discharged 36 days earlier. They required significantly fewer physiotherapy sessions (18.72 vs 46.49, p = 0.0009) resulting in a median reduction in total rehabilitation cost of £4982 (p = 0.0002) per patient. The total cost of additional investigations was £227 lower (p = 0.0369). Overall, the median cost without thrombectomy was £39,664 per case vs £22,444, resulting in median savings of £17,221 (p = 0.0489).

**Conclusions:**

Mechanical thrombectomy improved patient outcome, reduced length of hospitalisation and, even without procedural reimbursement, significantly reduced cost to the thrombectomy providing hospital.

## Introduction

Stroke is a leading cause of death and disability in the UK [1]. By 2035, the number of stroke cases in the UK is expected to increase by 44%, outpacing other European countries [2]. The total cost of health and social care for patients with acute stroke each year in the UK is £3.6 billion in the first 5 years after admission [3]. The economic costs of stroke in the UK from a societal perspective total around £9 billion a year which includes informal care costs like lost income due to care, benefit, disability and death [4].

For the management of acute ischaemic stroke, it has been shown that mechanical thrombectomy (MT) is associated with a shift towards better outcomes across the entire spectrum of disability as compared to conventional treatment alone (intravenous alteplase), with a number needed to treat to lower the outcome on the modified Rankin Scale (mRS) scale by one point as low as 2.6 [5].

The health-economic benefit of treating stroke is well established [6, 7]; however, no study so far has performed matched pair patient-level costing, examining immediate hospital costs associated with MT in comparison to conventional treatment. We focused on the first hospital episode and first 90 days post-event.

## Materials and methods

### Study design and patient selection

A matched-pair (1:1) cohort study design was used. From our prospective local stroke database 25 consecutive patients receiving MT were matched to 25 patients receiving conventional treatment regarding clinical and imaging data.

To compare hospital cost we used coarsened exact matching (CEM) and adjusted regression analysis, neglecting outcome [8]. This two-step approach is less prone to model misspecification and more robust than results based on the full unmatched data set [9].

The CEM process was based on: occluded vessel, lesion in dominant/non-dominant hemisphere, NIHSS at admission, ASPECTS score (assess by a neuroradiologist assisted by e-Stroke Suite, Brainomix Ltd, Oxford, UK) [10–12], age, pre-event mRS and co-morbidities (including atrial fibrillation, lipidaemia, diabetes mellitus, hypertension, previous stroke and chronic heart disease).

Written informed consent and formal ethical approval was not sought as the retrospective analysis of the data lack any treatment influence and were part of a service audit.

### Outcome data

Time from end of imaging (end of CT-Angiography) to arterial puncture was recorded, as well as the start of intravenous treatment (IVT). The occurrence of serious adverse events (SAEs) was evaluated from the medical records. Malignant oedema was defined as death caused by brain oedema following stroke. Symptomatic intracerebral haemorrhage (sICH) was defined according to the ECASS-II trial [13]. Three-month outcome was assessed by telephone interview, during an outpatient visit or by the community nurse using mRS [14].

### Cost analysis

To assess costs to the hospital we quantified the cost of hospital stay (length of stay (LoS) and level of care), cost of personnel, cost of thrombectomy, cost of additional procedures and cost of additional imaging.

#### Length of stay

The overall LoS was calculated as the time spent after the acute stroke or because of readmission within the first 90 days. To estimate the cost per bed day using NHS reference costs, the number of finished consultant episodes (FCEs), average unit costs and LoS associated to the main healthcare resource groups (version 4 HRG4 for stroke care excluding haemorrhages and other cerebrovascular accidents) were extracted. A weighted average by FCEs was then calculated across the HRG4 to estimate the unit cost for a bed day in a stroke unit [15]. Days spent in other Trusts were assumed at an average bed price of £251 [15]. Costs-per-bed at the admitting hospital were £838 for Intensive Care Unit with 2-organ support, £329 for Intensive Care Unit without organ support, £329 for High Dependency Unit, £277 for Acute Stroke Unit, £374 for Specialist Rehabilitation Ward, £251 for another hospital ward.

#### Cost for physiotherapy (PT), occupational therapy (OT), speech and language therapy (SALT)

A senior physiotherapist, occupational therapist and speech and language therapist assessed each patient and took into account their individual therapy needs in regard to neurological impairment and condition. Physiotherapy hours were calculated at 45 min/day on 5 days/week [15]. Therapists salary were calculated for a Grade 6 Therapist [16] (Table 1).

**Table 1:** Staff costing

	Cost
Surgeon/Interventional Radiologist	£321
Radiographer	£141
Circulation nurse	£135
Instrument nurse	£135
Anaesthesist	£321
Anaesthetic nurse	£135
Total staff cost (3 h)	£1188
Aspiration Kit Total	£1726
Other Kit	£590
Total instrument cost	£2316
Total cost of procedure	£3504

Table shows staff costing according to Curtis et al. [16] and Instrument costs according to industry pricing at the time of evaluation. Other Kit includes wire-1 £110, wire-2 £50, contrast £60, sheath £40, closure device £110, fluids £20, drape kit £95, catheter £105

#### Cost of thrombectomy procedure

Cost of thrombectomy was calculated at £3504. Staff costs were calculated for a 3 h procedure based on a microcosting approach [17, 18]. The mean basic salary for all staff, including consultant anaesthetist and surgeon, was taken from the Electronic Staff Record (ESR) [19]. This includes salary on-costs (employer’s national insurance plus 14.38% of salary for employer’s contribution to superannuation), qualification costs [20, 21]. Overheads were 24.2% of direct care salary costs and included administration and estates staff; non-staff costs were 43.1% of direct care salary costs and included costs to the provider for drugs, office, travel/transport, publishing, traning courses and conferences, supplies, clinical and general services, utilities such as water, gas and electricity [22]. Capital overheads (based on the new-build and land requirements of NHS hospital facilities) included accommodation for night-time dutie [23, 24]; working hours were calculated by deducting sickness absence days and study leave as reported for NHS staff groups [25] (Table 1).

Radiographer and Nurse costs were for Band 6 level [16]. Instrumentation including the cost of the sheath, catheter and other materials were cost based on industry pricing. The cost for alteplase was not included in the analysis, as it would usually be given to both groups.

#### Cost for additional examinations

Additional examinations and their cost were recorded for both groups. Initial baseline imaging (CT, CTA, CTP) was excluded as it applied to both patient groups as standard of care.

#### Statistical analysis

Baseline statistics were compared between groups using the Student’s t-test or the Chi-squared test. Where possible, outcomes were analysed using parametric analyses that adjusted for the matching variables depending on the type of data; this was linear regression for continuous variables, logistic regression for binary variables, and ordinal logistic regression for ordinal variables. In the case of non-normal continuous data, a logistic transformation was attempted to achieve normality. If these failed, non-parametric methods were used. A two-sided significance level of 0.05 was assumed for all analyses performed using Stata 14.

## Results

### Patient characteristics

The study involved 50 acute stroke patients that were considered for treatment. There was no significant difference in baseline characteristics between the two groups. Patient characteristics are listed in Table 2.

**Table 2:** Patient characteristics at baseline

Variable	Thrombectomy (N = 25)		Control (N = 25)		p-value
Age—mean (standard deviation)	66.96 (12.62)		68.68 (12.23)		0.627
Males	10 (40%)		13 (52%)		0.395
Co-morbidities	**Yes**	**No**	**Yes**	**No**	
Diabetes mellitus	14 (63.64%)	8 (36.36%)	15 (75%)	5 (25%)	0.426
Hypertension	11 (44%)	14 (56%)	9 (36%)	16 (64%)	0.564
Atrial fibrillation	15 (60%)	10 (40%)	14 (56%)	11 (44%)	0.774
Ischaemic heart disease	6 (24%)	19 (76%)	6 (24%)	19 (76%)	1.000
Congestive cardiac failure	3 (12%)	22 (88%)	8 (32%)	17 (68%)	0.088
Previous stroke	2 (8%)	23 (92%)	4 (16%)	21 (84%)	0.384
Dyslipidaemia	8 (32%)	17 (68%)	11 (45.83%)	13 (54.17%)	0.320
High body mass index	2 (8%)	23 (92%)	2 (8%)	23 (92%)	1.000
Neurological status					
NIHSS	17 (10–23)		16 (10–20.5)		0.410
Pre-mRS	25 (100%)		25 (100%)		0.274
0	22 (88%)		19 (76%)		
1	1 (4%)		5 (20%)		
2	1 (4%)		0 (0%)		
3	1 (4%)		1 (4%)		
Neuroradiological status					
ASPECTS/e-ASPECTS	9 (7.5–10)		10 (8.5–10)		0.361
5	0 (0%)		1 (4%)		
6	0 (0%)		1 (4%)		
7	5 (20%)		2 (8%)		
8	2 (8%)		1 (4%)		
9	5 (20%)		3 (12%)		
10	9 (36%)		13 (52%)		
Site of occlusion					1.000
M1	16 (64%)		16 (64%)		
M2	4 (16%)		4 (16%)		
Carotid-T	1 (4%)		1 (4%)		
Basilar artery	4 (16%)		4 (16%)		

Data for dyslipidemia was not available for 1 patient in the control group; for diabetes mellitus for 3 patients in the thrombectomy and 5 patients in the control group. Data presented as median (interquartile range) or number (percetange), unless indicate otherwsie

*N* number, *NIHSS* National Institute of Health Stroke Scale, *mRS* modified Rankin Scale, *ASPECTS* The Alberta Stroke Programme Early CT score, *e-ASPECTS* electronic ASPECTS, *M1* first segment of the middle cerebral artery, *M2* second segment of the middle cerebral artery

### Clinical outcome

**Fig. 1:**
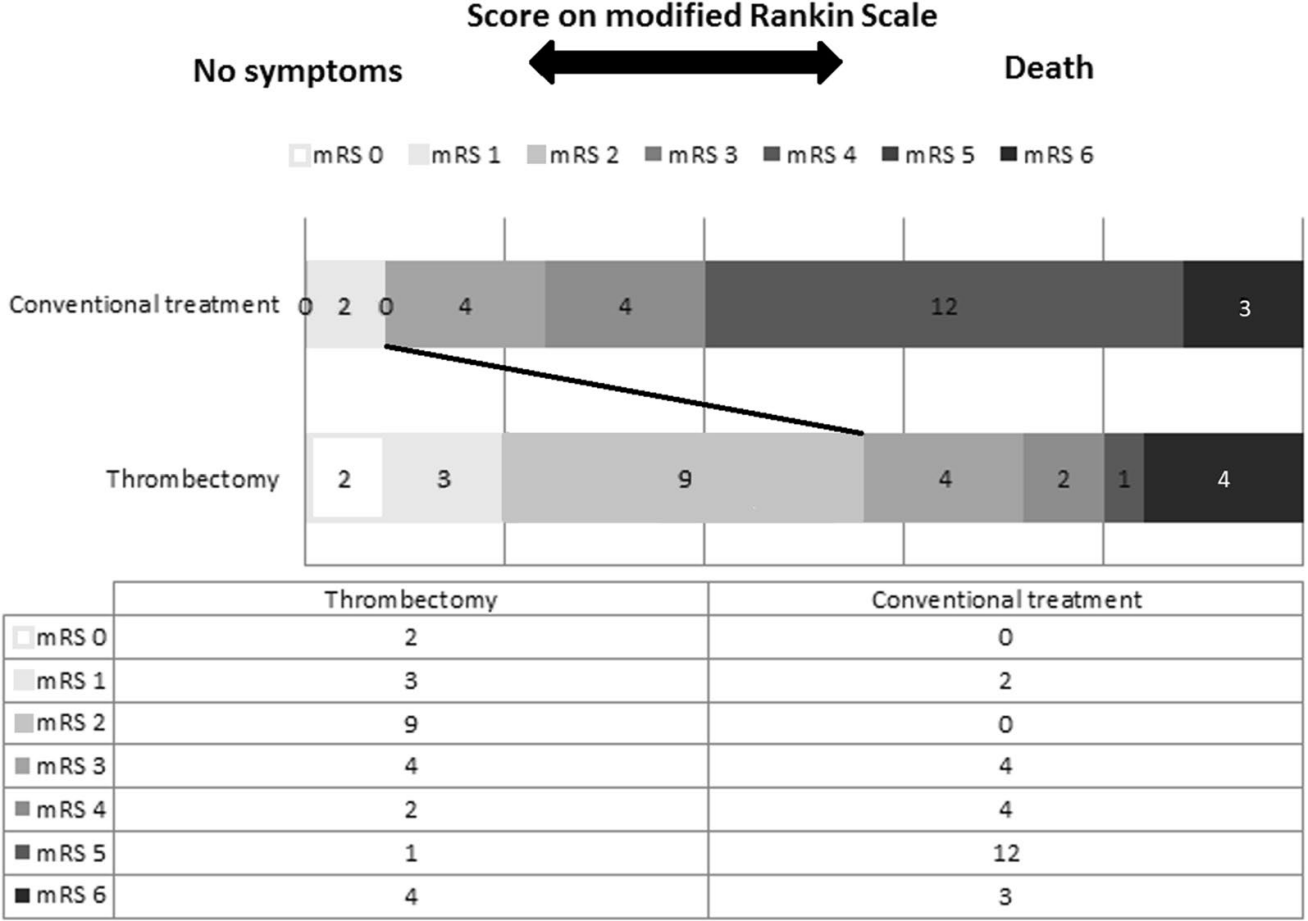
Clinical outcomes. This figure shows the distribution of functional scores at 90 days for patients in the thrombectomy group and the control group. Scores on the modified Rankin Scale (mRS) range from 0 to 6, with 0 indicating no symptoms and 6, death. Scores of 0–2 were combined for the analysis. A significant difference between the thrombectomy group and the control group was noted

Ordinal analysis of mRS was undertaken on the full range (0–6) of the mRS. The proportions of mRS 0–2 (good outcome) was compared between thrombectomy and control group (Fig. 1). Outcome on the mRS was adjusted for age, sex and NIHSS. Patients in the thrombectomy cohort had significantly more functionally independent ‘good outcomes’(56% vs 8%, p = 0.001) than the control group. There were significantly more patients with a very poor outcome of mRS 5–6 in the control group (60% vs 20%, p = 0.006). Patients that received thrombectomy were less likely to be wheelchair dependent (4% vs 48%, p = 0.005) when adjusted for age, sex, NIHSS. Significantly fewer patients remained in the most desolate state (mRS 5) when receiving thrombectomy (4% vs 48%, p = 0.005) (Fig. 1).

More patients in the thrombectomy group could be discharged back to their own homes (60% vs 48%), fewer were discharged to nursing homes (4% vs 16%), residential homes (0% vs 12%), rehab centres (8% vs 20%). There was no significant difference in the mortality rate between the 2 groups (16% vs 12%, p = 0.68), however, for mortality, the sample size was small.

Patients in the thrombectomy group had fewer serious adverse events in comparison to the control group (n = 30 vs 86, respectively). In terms of procedure-related complications for thrombectomy, there was one asymptomatic arterial dissection. Frequent serious adverse events are summarised in Table 3.

**Table 3:** Distribution of most frequent serious adverse events

Serious adverse events	Thrombectomy	Control
Symptomatic ICH	0	0
Asymptomatic ICH	4	1
Urinary tract infection	8	11
Pneumonia (including aspiration pneumonia)	5	8
Decompressive surgery due to cerebral oedema	4	2
Nosocomial infections	0	3
Pressure ulcer	1	3
Pulmonary embolism	0	2
Sepsis	0	2
Delirium	0	2

*ICH* intracranial heamorrhage

### Cost analysis

#### Cost due to LoS

For statistical analysis, log transformation was used to address the highly skewed LoS data. Using the median LoS, patients in the control group stayed significantly longer (p = 0.006). Overall, patients receiving thrombectomy spent an average of 44.44 (IQR 8–42) in hospital as opposed to an average of 80.24 (IQR 41–83) in the control group. There was a significant reduction in the number of days on Intensive Treatment Unit (ITU) or high dependency unit (HDU) (Table 4). The total overall bed day cost (all hospitals) for the thrombectomy group was £16,469 as opposed to the £31,984 in the control group (p = 0.0184; Table 3). The average bed day stay in the Thrombectomy hospital was 25.24 in the thrombectomy group and 65.24 in the control group (p = 0.0005) with a cost of £9288 as opposed to £26,270 in the control group (p = 0.0014). On average, patients in the thrombectomy group were discharged 36 days earlier with a cost savings of £15,516.

**Table 4:** Hospital length of stay

	Thrombectomy (mean)	Control (mean)	Difference (including deaths) p-value	Difference (excluding deaths) p-value
Bed days (Total, any Trust)	44.44	80.24	0.045	0.006
Bed days (Thrombectomy centre)	25.24	65.24	0.005	0.0005
Bed days (ITU/HDU)	1.84	6.92	0.0417	0.0306

*ITU* Intensive treatment unit, *HDU* high dependency unit

#### Excess bed days

Hospital stay beyond a pre-defined trim-point (nationally calculated average hospital stay for stroke patients set by the Department of Health and Social Care as 27 days) is referred to as excess bed days [26]. Overall, a greater number of patients in the thrombectomy group were discharged before the trim-point, and fewer patients in this group accounted for the excess bed days. In the thrombectomy group, 8 patients had hospital stays exceeding 27 days resulting in an additional 255 bed days added to the inpatient costs. This cost is substantially lower than the additional 959 bed days required by the 17 control patients. Furthermore, 13 of the 25 thrombectomy patients were discharged before the 27 days trim-point, sparing the hospital 237 bed days. Conversely, in the control group, only 4 patients were discharged before 27 days resulting in only 61 bed days spared.

#### Cost of in-hospital rehabilitation

Patients in the thrombectomy group required significantly fewer physiotherapy sessions (18.72 vs 46.49, p = 0.0009; Table 5). Patients in the control group required 42 h more physiotherapy, 23 h more speech and language therapy (SALT), 45 h more occupational therapy (combined 110 h). This accounts for £4982 of additional rehabilitation cost per patient. Approximating total hours worked by an Allied Health Professional per year at 1599 h, this equates to one post reduced for every 13 patients managed with thrombectomy.

**Table 5:** Overall cost comparison per patient

	Thrombectomy (mean)	Control (mean)	Difference (Control − Thrombectomy) (medians)	p-value
Physiotherapy sessions	18.72	46.49	27.77	0.0009
Physiotherapy time (min)	1215	3770	2555	0.0004
SALT time (min)	578	1957	1379	0.00005
Occupational therapy time (min)	1062	3770	2708	0.0001
Total rehabilitation time (min)	2854	9497	6643	0.0002
Total rehabilitation cost (£)	2141	7123	4982	0.0002
MT-trust bed cost (£)	9288	26,270	16,982	0.0014
Other trust bed cost (£)	7181	5715	− 1466	0.9778
Combined bed cost (£)	16,469	31,985	15,516	0.0184
Total cost of additional investigations (£)	330	557	227	0.0369
Total cost without procedure (£)	18,940	39,665	20,725	0.0127
Total cost with procedure (£3504)	22,444	39,665	17,221	0.0489

Above: Rehabilitation cost; Middle: Bed cost and additional investigations; Below: Total cost without and with MT procedure cost

*SALT* speech and language therapy

#### Cost for additional examinations

Patients in the control group needed nearly twice as many (112 vs 213) additional imaging examinations, mainly performed for adverse events. Per patient, control group patients had more investigations (8.9 vs 4.5, p = 0.0053). Control group patients also had more X-rays (5.0 vs 1.8, p = 0.0073) (Table 6).

**Table 6:** Additional imaging

Additional examination	Cost/examination (£)	Thrombectomy (N = 25)		Control (N = 25)	
		Number	Cost (£)	Number	Cost (£)
CT		40	3427	42	3464
CT-Head	71	33	2343	32	2272
CT-Abdomen + Pelivs	100	3	300	8	800
CT-Chest + Abdomen + Pelvis	196	4	784	2	392
MR	314	3	942	8	2512
X-ray	46	44	2024	124	5704
Doppler/Duplex	48	4	192	7	336
Abdominal ultrasound	40	1	40	6	240
Transthoracic echocardiogram	64	15	960	16	1024
Transoesophageal echocardiogram	118	1	118	1	118
Video swallow	78	3	234	6	468
Total		112	8251	213	13,866

*CT* computed tomography, *MR* magnetic resonance

### Projected cost savings

Cost savings were calculated at £17,221 per patient by taking the difference between mean total cost with conventional therapy and mean total cost with thrombectomy, which would equate to roughly £1,7 million for a centre with 100 mechanical thrombectomies per year.

## Discussion

Clinically, thrombectomy is intuitively attractive due to the demonstrated high rates of good clinical outcome with a low number needed to treat [5]. For the hospital, however, the first treatment is associated with an initial high cost to cover the thrombectomy procedure. This study looks at the immediate hospital cost associated with endovascular treatment, focusing on the first hospital episode and first 3-months post-event in patients with large vessel occlusion as compared to conservative medical therapy. Previous studies taking into consideration an NHS economic perspective were based on simulated Markov models with the data originating from different countries [27, 28]. To our knowledge, this is the only study to date analysing matched pair patient-level costs of thrombectomy in an NHS Trust for the admitting hospital as compared to conventional treatment. As there is level 1 evidence for endovascular stroke treatment in patients with LVO, primary randomisation was not appropriate on the basis of ethical concerns [29, 30].

Thrombectomy is known to be associated with a shift towards better outcomes across the entire spectrum of disability [5], which was also demonstrated in our patients, where clinical outcome was significantly improved in the thrombectomy group with a greater number of patients with functional independence (56% vs 8% p = 0.001), fewer serious adverse events (n = 30 vs 86) and fewer additional investigations (4.5 vs 8.9 per patient; p = 0.0053; n = 112 vs 213).

Thrombectomy patients could be discharged significantly earlier (mean 44 vs 80 days; p = 0.006) and were more likely to be discharged home (60% vs 28%). This is in line with Campbell et al. who reported on 70 patients from the EXTEND-IA study (35 in each arm, mean age 69, median NIHSS 15) where in the first 90 days thrombectomy patients spent significantly more time at home (median 73 days vs 15 days; p = 0.001). LoS in an acute stroke unit was reduced from mean 12 (control group) to 8 days (endovascular group), p = 0.04. Interestingly, there was no increase in intensive care time (p = 0.51) [31], whilst in our study we found a significant decrease in ITU/HDU time (mean 1.8 vs 6.9; p = 0.0306) if patients received thrombectomy. Mean LoS was significantly shorter both when including and excluding deaths.

In EXTEND-IA patients, rehabilitation LoS was reduced in the endovascular group (mean 33 vs 14 days), p = 0.03 [31]. The same was observed in our patient cohort. Patients in the control group required significantly more rehabilitation time (p = 0.0002) which accounted for £4982 of additional rehabilitation cost per patient. A recent publication by the Council of Deans for Health has highlighted that staff shortages in rehabilitation are putting health and social care services under pressure, with England currently facing one of its most profound and sustained workforce crises in decades [32].

Approximating 1599 total hours worked by an Allied Health Professional per year, this equates to one post made available to support the shortage of healthcare staff for every 13 patients managed with thrombectomy.

Several studies have demonstrated cost-effectiveness and, in many cases, cost savings with endovascular thrombectomy [33–37]. A recent study estimated costs for patients receiving endovascular therapy at different time points in an NHS setting but did not compare to a control group [38].

In the EXTEND-IA study, modelled life expectancy was calculated to increase by more than 4 years in the thrombectomy group with a significantly reduced loss of disability-adjusted life years and a clear gain of 4.4 quality-adjusted life years, translating to 90-day inpatient cost savings of US $14,880 [31]. Based on simulation modelling of 90-day mRS scores, Campbell et al. predicted a sustained and statistically significant mortality benefit up to 15 years post-treatment with associated benefits in DALYs lost and QALYs gained [31].

The THRACE trial described a probability of cost-effectiveness of additional thrombectomy treatment of 84.1% for cases with an averted disability and 92.2% regarding quality-adjusted life years. Additional costs per patient with averted disability were approximately 50% below the willingness to pay threshold [37]. Further analysis showed similar results with an overall cost-saving, even when considering the initially higher treatment costs [33, 36]. Previous studies have also reported on the long-term health economic benefit when patients with large vessel occlusion (LVO) are treated with thrombectomy [34, 35], demonstrating cost-effectiveness for all subgroups of patients undergoing mechanical thrombectomy, except for those with ASPECTS < 5 and M2 occlusion-where data has so far been scarce [39]. A recent meta-analysis based on 23 studies concluded that the addition of mechanical thrombectomy is cost-saving for a patient between 50 and 79 years and cost-effective for patients between 80 and 100 years [40]. Menon et al. found that even patients with proximal M2 segment middle cerebral artery (MCA) occlusion benefitted from mechanical thrombectomy [41].

In our study, cost savings to the admitting/treating hospital were calculated at £17,221 per patient by taking only the difference between care bed days, additional investigations and rehabilitation. Importantly, no reimbursement for devices or the thrombectomy procedure was added. Fixed costs as per NHS reference costs were used, based on the number of speciality bed hours, (ITU, High Dependency Unit, Hyperacute Stroke Unit) and other standard ward bed days [15]. As patients with similar number of bed days required different intensity of management, we captured imaging, nursing and allied health interventions which otherwise may lead to variation in true patient costs. Additional costs for treatment of serious adverse events such as pulmonary embolism, sepsis, pressure ulcer, urinary tract infection, decompressive surgery, nosocomial infections and their medication were not included as they are reflected in the LoS and subject to the local payment system (i.e.block contract).

In the thrombectomy group, only 8 patients (32%) had hospital stays exceeding the trim-point, resulting in an additional 255 bed days added to the inpatient cost, versus more than twice as many (n = 17; 68%) in the control group, adding 959 additional bed days. Benefits of a shorter LoS include patient well-being with a lower risk of hospital-acquired infections and an increase in hospital capacity for new admissions and increased availability of ITU beds.

A strength of this study is its analytical approach, minimising bias due to different covariates. If covariates differ between groups, the results of regression analysis alone can be misleading [8], which is why we used coarsened exact matching (CEM), neglecting outcome and, as a next step performed adjusted regression analysis to account for the remaining bias in co-variants, again neglecting outcome [8]. This two-step approach is reported to be less prone to model misspecification and even more robust than results based on the full, unmatched data set [9, 42, 43]. Another strength is that data was collected for each patient, capturing individual levels of required care, physiotherapy, speech, language and occupational therapy.

A limitation of our study is its retrospective analysis and the relatively small sample size which, nevertheless clearly demonstrated significance on a 95% confidence interval. Another limitation is that any bias of omitted covariates cannot be completely eliminated. Also, we did not perform a baseline severity-adjusted endpoint analysis as previously suggested by Saver et al. [44]. However, pre-mRS was matched, and there was no significant difference between groups.

We couldn't assess long-term community care costs or calculate QALY's to compare the costs with the willingness to pay threshold as outcome past 90 days was not assessed. However, given the significantly greater level of disability in the control group, the costs of care beyond 90 days is expected to remain greater than in the endovascular group.

The cost of providing secondary ambulance transfers in cases where patients needed to be transported from a general hospital to the thrombectomy-capable hospital was not included in the calculated as it did not affect the thrombectomy-capable hospital. It would, however, provide an additional argument for establishing more thrombectomy-capable hospitals.

## Conclusion

For the admitting hospital, thrombectomy makes sense financially and clinically, independent of additional reimbursement for the procedure. Thrombectomy reduced disability and LoS, leading to significant cost savings by 90 days. If these cost savings are extrapolated to all eligible stroke patients, the benefits would substantially lessen the economic burden of the entire healthcare system.

## Data Availability

The datasets used and/or analysed during the current study are available from the corresponding author on reasonable request.

## Abbreviations

UK: United Kingdom
MT: Mechanical thrombectomy
mRS: Modified Rankin Scale
CEM: Coarsened exact matching
NIHSS: National Institutes of Health Stroke Scale
ASPECTS: Alberta stroke programme early CT score
CT: Computed tomography
IVT: Intravenous treatment
SAEs: Serious adverse events
sICH: Symptomatic intracerebral haemorrhage
LoS: Length of stay
NHS: National Health Institue
FCEs: Finished consultant episodes
PT: Physiotherapy
OT: Occupational therapy
SALT: Speech and language therapy
ESR: Electronic Staff Record
CTA: Computed tomographic angiography
CTP: Computed tomographic perfusion
IQR: Interquartile range
ITU: Intensive Treatment Unit
HDU: High dependency unit
LVO: Large vessel occlusion
MCA: Middle cerebral artery
